# A lexicon-based approach to examine depression detection in social media: the case of Twitter and university community

**DOI:** 10.1057/s41599-022-01313-2

**Published:** 2022-09-21

**Authors:** Junyeop Cha, Seoyun Kim, Eunil Park

**Affiliations:** 1 Department of Applied Artificial Intelligence, Sungkyunkwan University, Seoul, Korea; 2 Department of Interaction Science, Sungkyunkwan University, Seoul, Korea

**Keywords:** Health humanities, Cultural and media studies

## Abstract

Globally, the number of people who suffer from depression is consistently increasing. Because both detecting and addressing the early stage of depression is one of the strongest factors for effective treatment, a number of scholars have attempted to examine how to detect and address early-stage depression. Recent studies have been focusing on the use of social media for depression detection where users express their thoughts and emotions freely. With this trend, we examine two-step approaches for early-stage depression detection. First, we propose a depression post-classification model using multiple languages *Twitter* datasets (Korean, English, and Japanese) to improve the applicability of the proposed model. Moreover, we built a depression lexicon for each language, which mental health experts verified. Then, we applied the proposed model to a more specific user group dataset, a community of university students (*Everytime*), to examine whether the model can be employed to address depression posts in more specific user groups. The classification results present that the proposed model and approach can effectively detect depression posts of a general user group (*Twitter*), as well as specific user group datasets. Moreover, the implemented models and datasets are publicly available.

## Introduction

Mental illness has a significant prevalence and burden globally. The World Health Organization (WHO) reported that there has been a 13% increase in mental illness patient occurrence in the recent decade. Among them, 2.8 billion people suffer from depression, one of the leading causes of disability and a significant contributor to the global illness burden (WHO, 2021a). In general, depression is defined as “*a series of mental health issues characterized by loss of interest and enjoyment in everyday life, low mood, and selected emotional, cognitive, physical, and behavioral symptoms*” (Collo and Pich, 2018).

Depression can be diagnosed by medical history, physical exam, lab tests, or psychological evaluation that answers questions about thoughts, emotions, and behavior. There are also many effective treatments for depression. Depending on the severity and pattern of depression, there are treatments such as behavioral activation, cognitive behavioral therapy, interpersonal psychotherapy, selective serotonin reuptake inhibitors, and tricyclic antidepressants. Through the methods mentioned before, depression can be diagnosed early, and early diagnosis is a critical factor in depression treatment (Conus et al., 2014).

Although depression can be treated early and effectively at a relatively low cost, the gap between those who can and cannot receive such available treatments is still significant. Some people, who suffer from depression, are not even aware of their condition and the need for treatment. Regarding issue is proven by the fact that only 4.6% of the world population suffer from depression, 43.3% of them do not take their symptoms seriously and do not care to be treated professionally (Thornicroft et al., 2017). The unawareness of depression treatment leads to failure of early diagnosis and treatment that is related to a longer illness duration and more relapses (Hunt and Eisenberg, 2010).

Several methods are proposed to help people who cannot receive adequate diagnosis and treatment. The Mental Health Gap Action Programme aims to improve services for mental, neurological, and drug use disorder care in low- and middle-income countries (WHO, 2021b). In addition, many simple questionnaires are available online that allow you to diagnose whether you are depressed or not without the help of experts. However, as some people who suffer from depression do not want to be disclosed (APA, 2021), the methods mentioned above are insufficient to find depression patients in blind spots.

With such a trend, taking action for plenary depression diagnosis has become one of the momentous research topics. It indicates that exploring new information sources for depression diagnosis should also be highlighted (De Choudhury and De, 2014). As one of the potential information sources, scholars have focused on social media, where users tend to be more straightforward and honest about their feelings and opinions. Moreover, several users often share their mental health issues and seek solutions to mental illness diagnoses and treatments (Shen and Rudzicz, 2017).

These behaviors allow scholars to use social media as one of the potential solutions in exploring both awareness and diagnosis of depression symptoms. For instance, Kim et al. (2020) using social media datasets is useful in detecting social media users’ emotional statements and potential mental illness.

However, the majority of depression-related research using social media has been conducted with an English dataset. It means that addressing depression issues with low-resource languages can be more challenging (Shen et al., 2018). To combat the language bias in the field, Bataineh et al. (2019) focused on Arabic as their main language to find depressed users on social media. Thus, we propose a deep learning framework that examines whether it is possible to detect users’ depression from three different language datasets: English, Korean, and Japanese, in Study 1. Specifically, we attempt to address the following research question (RQ):

- RQ 1: Can we examine whether a user’s post on social media represents depression?

Related to our first RQ, there can be a doubt that the presented classification approach is effective and useful for a specific user group, such as a university community. Young adults who are suffering from mental health issues significantly increased over the last decade (Zhao et al., 2020), and the number of undergraduate students who suffer from depression is consistently increasing globally (IHME, 2021). Because of the emotional dynamics of the younger generation, it is required to closely examine the incidence of depressive symptoms in the younger generation (Ochnik et al., 2021). According to the Student Experience in the Research University (SERU) Consortium survey, the COVID-19 pandemic has been reported to have a negative impact especially on the mental health of undergraduate students (Chirikov, 2020). Furthermore, it has been shown that this kind of additional stress due to COVID-19 can degrade their learning experience (Kecojevic et al., 2020). Undergraduate students are typically more vulnerable when facing this kind of pandemic situation due to their lack of resources to cope with it and experience high levels of stress, anxiety, and depression. Thus, we wish to address the issue with the following RQ in Study 2:

- RQ 2: Can we identify whether an undergraduate student’s depression posts on his/her university community?

In this study, we developed a deep learning-based prediction model for the early detection of high-risk groups of depression, which is a major social problem worldwide, using social media data. In addition, we confirmed the efficiency and usefulness of the model, using an online community for university students to predict high-risk groups for depression among university students who have been greatly affected by COVID-19.

## Related work

### Identifying depression in social media

It has become a norm for users to post their feelings and activities on social media. This notion allows scholars to explore each user’s mental health issues projected by their activities and behavior on social media, and these actions can be a chance to capture their mental state or conditions (Lee et al., 2020).

In accordance with such a trend, there has been a huge amount of interest in detecting a user’s depression, based on several information sources of social media (e.g., posts, images) through a computational framework. Pirina and Çöltekin (2018) conducted a series of experiments to examine depression *Reddit* post features with a support vector machine (SVM) approach. Orabi et al. (2018) attempted to classify the depression posts on *Twitter* through text differences between control and depressed groups with two bench-marking datasets, CLPsych2015 (Coppersmith et al., 2015) and Bell Letters Talk datasets using both convolutional neural network (CNN) and recurrent neural network (RNN) models. Recently, Zogan et al. (2021) examined an automatic depression detection task by fusing two asymmetric parallel networks (user behavior and user post history network), which are organized by CNN and gated recurrent units model (GRU).

To facilitate computational approaches, some cornerstone research has been presented. De Choudhury et al. (2013) proposed and validated highly frequent depression-oriented unigrams in *Twitter* with four themes: Symptoms, Disclosure, Treatment, and Relationships life. Tadesse et al. (2019) provided a number of linguistic features (from sentimental analysis, LDA, uni- and bi- grams), which allowed researchers to address users’ depressed attitudes on one of the topic-oriented social media channels, *Reddit*.

It indicates that users’ contents on social media have a high probability of containing distinctive markers and signals for identifying users’ mental health status and illnesses (e.g., depression).

### Depression of undergraduate students

Due to the COVID-19 pandemic, which posed both restrictions on outdoor activities and reductions in personal income, the prevalence of depression has increased in a number of countries. In specific, many countries reported that the incidence rate of depression among young generations is significantly greater than that of the general population (OECD, 2021).

Thus, the prevalence of depression in young generations has been recently highlighted as a social issue. Islam et al. (2020) investigated the prevalence of depression and anxiety on 476 Bangladeshi undergraduate students through cross-sectional web-based surveys. Based on the survey results, they found that the symptoms of depression and anxiety of 392 and 389 students appeared from mild to severe levels.

Ochnik et al. (2021) also conducted cross-national (Colombia, the Czech Republic, Germany, Israel, Poland, Russia, Slovenia, Turkey, and Ukraine) research about the mental health problems of undergraduate students, including anxiety and depression, during the COVID-19 pandemic. They found various risk factors of depression for each country and argued that both social and cultural backgrounds should be considered in addressing mental health problems in the student population. Moreover, Zhao et al. (2020) compared depression symptoms of 821 undergraduate students in South Korea, China, and Japan, and found notable mental health issues, which are crucially related to the COVID-19 pandemic, through online questionnaire items (Patient Health Questionnaire-9).

Although prior studies showed significant implications in undergraduate students’ depression with consideration of the COVID-19 pandemic, the majority of the research has employed survey-oriented approaches, which can be difficult to detect the students’ depression. Moreover, only a limited amount of attention has been presented to whether low-resource language dictionaries, Korean and Japanese, can be useful for examining depression detection with consideration of both common social media users and undergraduate students. Thus, this paper introduces and evaluates a framework for examining depression detection using several depression dictionaries created from English, Korean, and Japanese social media datasets.

## Study 1: general depression classification model in social media

**Fig. 1:**
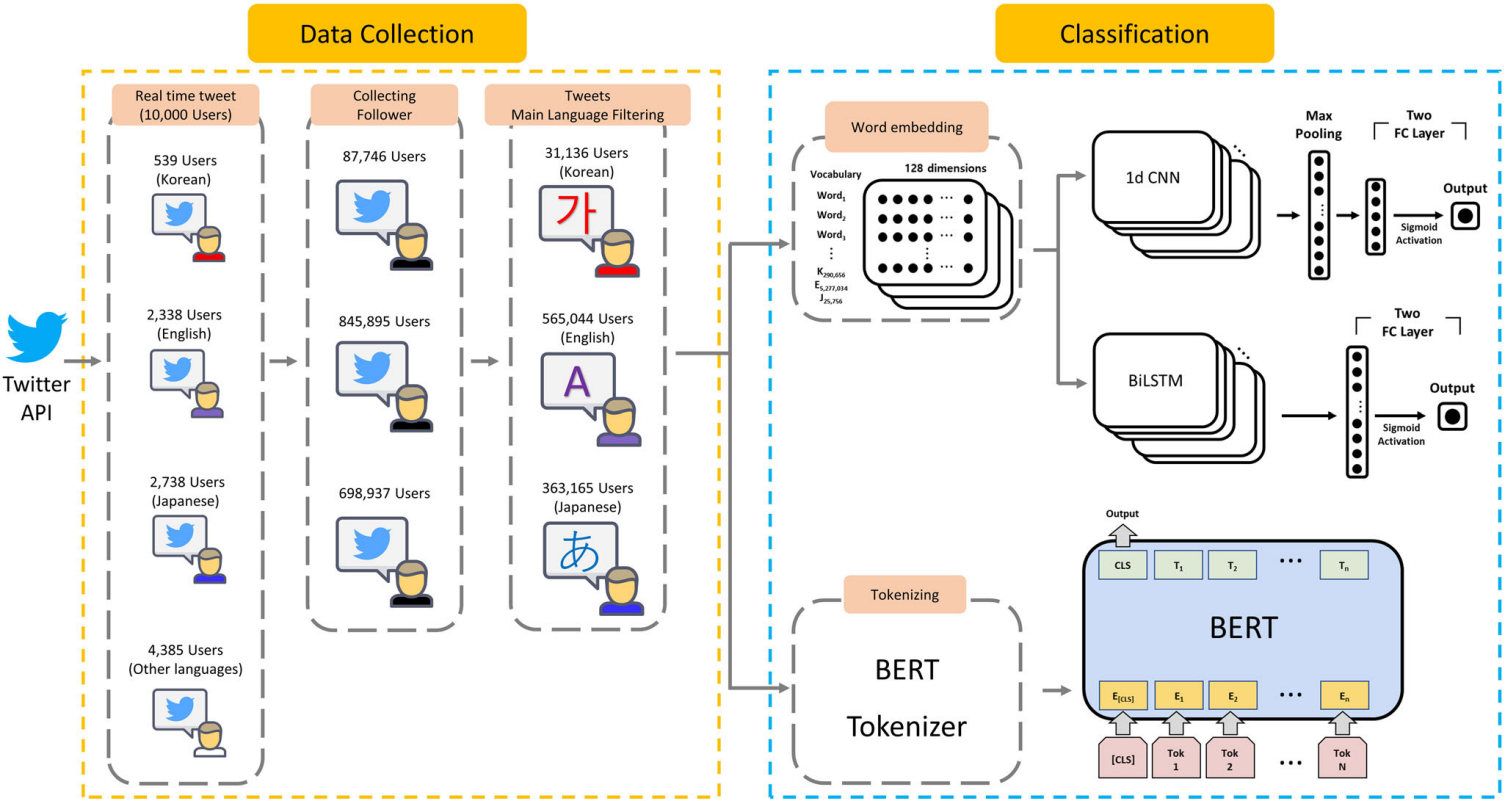
An workflow of data collection and architecture of the proposed CNN, BiLSTM, and BERT-based classification model.

The workflow and overview of Study 1 are presented in Fig. 1. The data collection procedures and classification model are examined in the following sections.

### Data collection

**Fig. 2:**
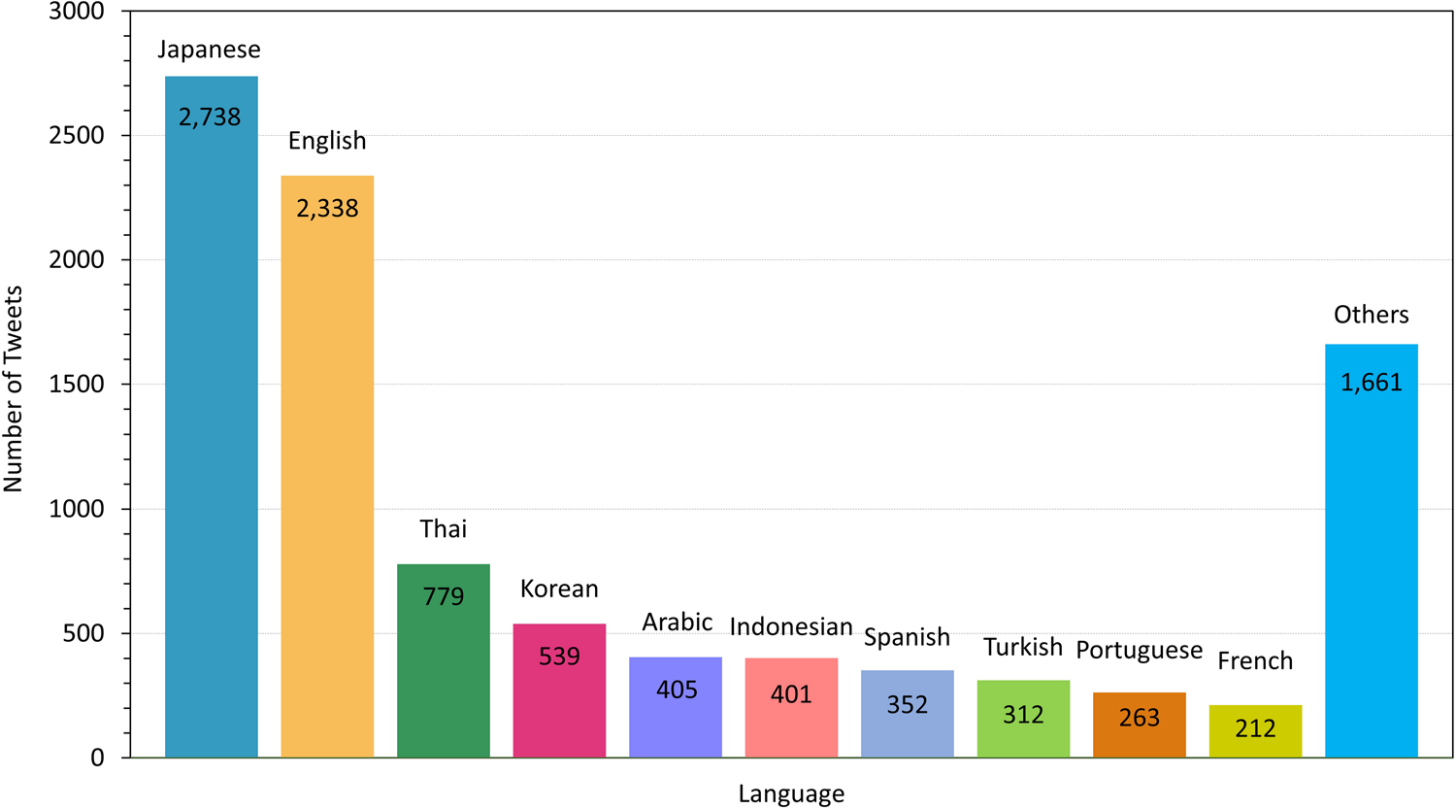
The main language distribution of 10,000 randomly collected users on Twitter.

To collect users’ posts on social media for depression detection, we used the *Twitter* APIs (Application Programming Interface). *Twitter* is a famous microblogging and social networking service where users post tweets and interact with messages. *Twitter* has 36 million active users, 500 million tweets are sent per day and 51.8% of users are in the 18–34 and 28.4% are in the 35–49 years old age bracket (Finance Online, 2022). We adopted a community-based random sampling approach (Zhang et al., 2019), which samples the posts from the followers of the current user to collect random user’s posts. We used *Twitter* sampled stream API, which crawls 1% of real-time posts until retrieving 10,000 unique user accounts. User accounts’ language distribution of the top 10 languages on *Twitter* is shown in Fig. 2. Then, we employed the accounts that had their main language set in Korean, English, or Japanese. To collect more randomized users, we crawled the collected user accounts’ followers. Because the followers’ main language can differ from the followed accounts, we conducted a main-language filtering procedure as follows. If a user’s one of five recent posts includes English, Korean, or Japanese, we defined the user’s main language as English, Korean, or Japanese, respectively. As a result, we collected 31 thousand, 565 thousand, and 363 thousand users whose main languages were Korean, English, or Japanese.

We conducted the user pre-processing procedure as follows. First, we excluded user accounts that posted none or more than 20 posts per day. Moreover, we excluded user accounts that present ‘moved to’ (Korean: , Japanese: ) as such accounts were no longer in use. We also excluded accounts with specific hyperlinks in their descriptions. After these procedures, we obtained about 14 thousand (Korean), 210 thousand (English), and 216 thousand (Japanese) user accounts. Thus, we collected up to 100 recent posts from each account and gathered 921 thousand (Korean), 10 million (English), and 15 million (Japanese) posts. All posts were created between Jan. 2019 and Mar. 2021. We also conducted preprocessing procedures on each post (i.e., removing email address, url, and contents of non-selected languages).

### Lexicon-based labeling

Because a lexicon-based approach is one of the efficient ways to handle large-scale text datasets, several scholars mainly employed it in addressing text-based tasks (e.g., sentimental analysis) (Mukhtar and Khan, 2020). Thus, we employed this approach to explore whether each post can be classified as a depression post. We also built multi-lingual depression lexicon lists through three procedures: collection, translation, and verification.

#### Lexicon collection

We reviewed three key prior studies that are significantly related to the intersection between social media and depression and collected core keywords for multilingual depression lexicon datasets (De Choudhury et al., 2013; Cheng et al., 2016; McCosker and Gerrard, 2021). The keywords related to depression, mainly proposed and introduced by prior research, were selected. After excluding duplicated keywords, 69 keywords remain as our lexicons. Then, because our lexicons were written in English, we translated each keyword to Korean and Japanese, respectively. We use Naver (PapagoLee et al., 2016), and Google Translate API (https://translate.google.co.kr/). Then, the translation results were reviewed, verified, and revised by the following procedures:

1. Three experts were asked to explore whether each keyword is ‘correct (0)’, ‘not correct (1)’, or ‘need to complement’ (2).
2. If the experts labeled a translated keyword as ‘not correct’ (1) or ‘need to complement’ (2), The experts were instructed to revise the keyword.
3. The experts were requested to review whether the revised keywords have the intended meaning.

For instance, because ‘stressed’ was translated as  (English: emphasized), the experts complimented it to  (English: stress out).

#### Lexicon verification

Because lexicon quality is one of the most significant determinants in lexicon-based text analysis and evaluations (Madkar et al., 2021), we verified whether each keyword in our lexicon dictionaries is mainly related to depression. Two researchers, who possess a master’s degree in psychology, evaluated whether each keyword is associated with depression with a 5-point Likert scale (5: significantly relevant). Then, the lexicons below 3 points were excluded. Table 1 shows our English, Korean, and Japanese depression lexicon dictionaries, composed of 31, 32, and 32 keywords, respectively.

**Table 1:**
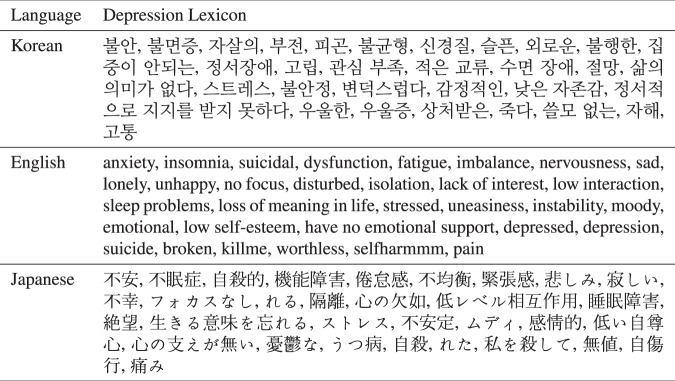
Depression lexicon for Korean, English, and Japanese.

#### Post Labeling

To label each post as depression or non-depression, we employed part-of-speech (POS) tagging to the posts and our depression lexicon dictionary. Because each language has its unique linguistic characteristics (e.g., no spacing in Japanese), we employed different POS taggers for each language. We used Khaiii (https://github.com/kakao/khaiii) (Kakao hangul analyzer) for Korean, the natural language toolkit (NLTK) (https://www.nltk.org/) for English, and fugashi for Japanese (McCann, 2020). After using POS taggers on posts and depression lexicon dictionary, we kept the following POS:

- Korean: ‘NNG’, ‘NNB’, ‘VV’, ‘VA’, ‘XR’
- English: ‘NN’, ‘NNS’, ‘JJ’, ‘VB’, ‘VBG’, ‘VBN’, ‘RP’, ‘DT’, ‘IN’, ‘TO’
- Japanese: , ,

Then, we labeled each post as a depression class when it included at least one depression lexicon.

#### Descriptive statistics

Based on these procedures, descriptive statistics of our final dataset are presented in Table 2.

**Table 2:** Data description for each language.

Language	Word count of posts (standard deviation)
Korean	8.02 (7.93)
English	13.22 (9.59)
Japanese	3.45 (3.09)

### Sampling method

To combat the data imbalance issues of social media datasets identified in prior depression research (Kim et al., 2020; De Choudhury et al., 2013), suitable sampling techniques are required to address these imbalance issues (Sharma and Verbeke, 2020). Thus, we employed SMOTE and under-sampling procedures (Shalizi and Rinaldo, 2013).

### Depression post classification

For the classification of the depression posts, we used three off-the-shelf baseline classification models as follows:

- 1-D CNN: The employed CNN model is organized by a number of layers, which include an embedding layer, convolutional layer, max-pooling layer, fully connected layers, and the output. The embedding layer, which is the first layer of the model represents the word features of a pre-processed post with 128 dimensions, and its weight is initialized by the pre-trained word2vec. Second, the convolutional layer with word embedding input consists of 128 filters, while each filter has a size of four. Furthermore, to avoid over-fitting problems, we used a dropout. The next layer is a max-pooling layer with a size of 128 that takes the maximum values from the CNN filters. The output of the max-pooling layer is passed through two fully connected layers. The final output is the probability of the classification through the sigmoid activation function, which ranges from 0 to 1. For training, we used the binary cross-entropy loss function and Adam optimizer.
- Bidirectional Long Short-Term Memory (BiLSTM): To employ a bi-directional LSTM classification model, we applied the same word-embedding procedures in the CNN classification model. The model architecture is organized by an embedding layer, BiLSTM layers, fully connected layers, and the output. The first embedding layer is the same as the CNN classification model. Second, the BiLSTM layer with word embedding input consists of 64 units. The following processes are the same as the CNN classification model.
- Bidirectional Encoder Representations from Transformers (BERT): To employ a BERT model, we adopted a different built-in BERT tokenizer, and BERT model for each language, respectively. We set the embedding dimension as 768 and the extracted feature was sent to one fully connected layer. We employed a cross-entropy loss function and AdamW optimizer. We used three BERT models, BERT for English, KoBERT (https://github.com/SKTBrain/KoBERT) for Korean, and tohoku-BERT (https://github.com/cl-tohoku/bert-japanese/tree/v1.0) for Japanese.

All experiments were conducted on a single Tesla V100 PCIE 32 GB GPU and implemented in Python 3.6. For each classification model, the overview of the employed architectures is presented in Fig. 1. The detailed configurations are shown in Supplementary Table A1.

### Evaluation metrics

Three evaluation metrics are employed to investigate whether our proposed models can classify the depression classes well: *precision*, *recall*, and *F1-score*.

### Result

Tables 3, 4 and 5 present the results of evaluation metrics. The BERT-based classification model with under-sampling methods reported the highest F1-score than the other baseline models, owing to its context-dependent embedding. In the normal sampling, however, F1-score is relatively low in all selected languages and models because of the class imbalance issues. Especially, because the Japanese dataset is the most class-imbalanced dataset, the performance of models on the Japanese dataset is relatively lower than other models. Moreover, the BERT-based classification models can be more vulnerable to class imbalance issues than other baseline models, CNN and BiLSTM.

**Table 3:** Results of the binary classification task on *Twitter* with normal sampling.

Sampling	Model	Language	Label	Accuracy	Precision	Recall	F1-score
Base	CNN	Korean	non-depression	0.9990	0.9994	0.9996	0.9995
			depression		0.9830	0.9763	0.9796
		English	non-depression	0.9984	0.9987	0.9997	0.9992
			depression		0.9448	0.7897	0.8603
		Japanese	non-depression	0.9992	0.9995	0.9997	0.9996
			depression		0.7213	0.6132	0.6628
	BiLSTM	Korean	non-depression	0.9991	0.9995	0.9995	0.9995
			depression		0.9815	0.9804	0.9809
		English	non-depression	0.9983	0.9988	0.9996	0.9992
			depression		0.9221	0.8058	0.8600
		Japanese	non-depression	0.9993	0.9996	0.9997	0.9997
			depression		0.7618	0.6676	0.7116
	BERT	Korean	non-depression	0.9787	0.9794	0.9992	0.9892
			depression		0.8145	0.1447	0.2457
		English	non-depression	0.9991	0.9995	0.9995	0.9892
			depression		0.9264	0.9262	0.9263
		Japanese	non-depression	0.9993	0.9996	0.9997	0.9997
			depression		0.7499	0.6623	0.7032

**Table 4:** Results of the binary classification task on *Twitter* with under-sampling.

Sampling	Model	Language	Label	Accuracy	Precision	Recall	F1-score
Under-sampling	CNN	Korean	non-depression	0.9893	0.9966	0.9894	0.9900
			depression		0.9966	0.9819	0.9892
		English	non-depression	0.9928	0.9948	0.9908	0.9928
			depression		0.9908	0.9948	0.9928
		Japanese	non-depression	0.9925	0.9874	0.9977	0.9925
			depression		0.9976	0.9873	0.9924
Under-sampling	BiLSTM	Korean	non-depression	0.9900	0.9835	0.9966	0.9900
			depression		0.9966	0.9833	0.9899
		English	non-depression	0.9984	0.9975	0.9922	0.9948
			depression		0.9922	0.9975	0.9949
		Japanese	non-depression	0.9925	0.9967	0.9984	0.9925
			depression		0.9984	0.9865	0.9924
	BERT	Korean	non-depression	0.9966	0.9986	0.9946	0.9966
			depression		0.9946	0.9986	**0.9966**
		English	non-depression	0.9965	0.9999	0.9931	0.9965
			depression		0.9932	0.9999	**0.9965**
		Japanese	non-depression	0.9939	0.9922	0.9956	0.9939
			depression		0.9956	0.9922	**0.9939**

Bold values represent the greatest levels.

**Table 5:** Results of the binary classification task on *Twitter* with over-sampling.

Sampling	Model	Language	Label	Accuracy	Precision	Recall	F1-score
Over-sampling	CNN	Korean	non-depression	0.9356	0.9254	0.9475	0.9363
			depression		0.9462	0.9236	0.9348
		English	non-depression	0.9572	0.9459	0.9699	0.9577
			depression		0.9691	0.9446	0.9567
		Japanese	non-depression	0.9643	0.9640	0.9647	0.9643
			depression		0.9647	0.9639	0.9643
	BiLSTM	Korean	non-depression	0.9465	0.9310	0.9645	0.9475
			depression		0.9632	0.9285	0.9455
		English	non-depression	0.9607	0.9438	0.9798	0.9614
			depression		0.9790	0.9417	0.9599
		Japanese	non-depression	0.9645	0.9560	0.9737	0.9648
			depression		0.9732	0.9552	0.9641
	BERT	Korean	non-depression	0.5000	0.0000	0.0000	0.0000
			depression		0.5000	1.0000	0.6667
		English	non-depression	0.9896	0.9812	0.9983	0.9897
			depression		0.9983	0.9809	0.9895
		Japanese	non-depression	0.5000	0.0000	0.0000	0.0000
			depression		0.5000	1.0000	0.6667

In general, the models with under-sampling methods show a greater F1-score than those with over-sampling methods, because our employed over-sampling methods, SMOTE, did not consider the current neighboring examples, which can be inferred by other classes. It may result in both class overlapping and noise issues. In the case of our BERT-based classification, as SMOTE did not consider special tokens of BERT, [CLS], and [SEP], several generated sequences include more than two [CLS] or [SEP] tokens. Especially, for Korean and Japanese, the BERT-based classification model predicted all samples as depression posts.

## Study 2: Undergraduate student depression detection model

We examined whether our depression classification approaches, which were trained and tested by our *Twitter* gathered depression lexicon dataset, can be applied to online communities of specific user groups in South Korea. We checked the scalability of the model while applying the model learned from *Twitter* data to another online community.

South Korea, in fact, has been hit hard by the COVID-19 outbreak and recorded the second largest number of confirmed cases of COVID-19 in the world at the end of February 2020 (Zhao et al., 2020). In addition, mental illness is estimated to cause deterioration of health and decreased production, resulting in an annual loss of about $2.5 trillion (WHO, 2021a) and social and economic losses due to mental illness, including depression, and behavioral disorders reach 7 trillion won a year (TLG Health, 2020).

Although there are a huge number of considerable user groups, we aim to apply our classification models to online communities of undergraduate students, whose mental health conditions are crucially affected by the COVID-19 pandemic (Lee et al., 2021).

### Online community of undergraduate students: *Everytime*

Before the outbreak of COVID-19, people hung out with their friends face-to-face. However, after the COVID-19 breakout in South Korea, due to the outdoor restriction policies implemented by the government, the majority of the universities have restricted their undergraduate students’ offline attendance on campuses. This indicates that most undergraduate students have less chance of exchanging information and communicating face-to-face with other students. Also, unlike before the outdoor restriction, most of the students lost their chance to spend time together with their friends outside. These restrictions affected the health of undergraduate students, especially their mental health (Mao, 2021).

However, because of the restriction, the online communities for undergraduate students, such as *campuspick*^1^ or *Facebook bamboo forest page*, have been continuously highlighted as places of information-exchange and communication. Among these communities, *Everytime* is the most active community in South Korea with 5.88 million student users^2^. *Everytime* is composed of different types of topic-oriented forum pages such as general, secret, and information forum pages.

To join the *Everytime* community of each university, each undergraduate student must submit his/her certificate of enrollment and verify his/her institutional email address. It means that faculty members, staff, and graduate students cannot join the community, while only undergraduate students can join the community. Moreover, because *Everytime* guarantees its undergraduate students’ anonymity, the students can freely discuss their concerns and share information. Therefore, the students are more likely to share their mental health problems (De Choudhury and De, 2014).

### Data collection

To make use of *Everytime*’s characteristics, we collected the students’ posts on two forum pages of *Everytime* community of one of the South Korean private universities: general and depression-related forum pages.

We collected and labeled the posts from the general (as non-depression posts) and depression-concerns forums (as depression posts) of one of the private universities in Korea. As a result, we collected both 16,681 non-depression and 5322 depression posts from August 10, 2018, to October 18, 2021. Then, all posts with lower than 10 words and unnecessary text contexts were excluded. After these procedures, the same number of depression posts was randomly selected, checked, and validated by three researchers. We ended up with 5203 non-depression and 5290 depression posts. Note that all users’ personal information was anonymized. All procedures were approved by the institutional review board of one of the national universities. Table 6 shows the descriptive statistics of the collected dataset.

**Table 6:** Data description of *Everytime* dataset.

Language	Word count of posts (standard deviation)
Korean	24.23 (54.52)

**Table 7:** Results of the binary classification task on *Everytime* with normal-sampling.

Sampling	Model	Label	Accuracy	Precision	Recall	F1-score
Based	CNN	non-depression	0.3732	0.3698	0.9926	0.5388
		depression		0.7195	0.0112	0.0220
	BiLSTM	non-depression	0.4956	0.4957	0.9956	0.6619
		depression		0.4651	0.0038	0.0075
	BERT	non-depression	0.5423	0.5202	0.9900	0.6820
		depression		0.9120	0.1019	0.1833
Under-sampling	CNN	non-depression	0.5149	0.5079	0.9576	0.6638
		depression		0.6299	0.0721	0.1294
	BiLSTM	non-depression	0.5091	0.5079	0.9521	0.6579
		depression		0.6091	0.0733	0.1309
	BERT	non-depression	0.7252	0.6514	0.9587	0.7757
		depression		0.9242	0.4955	**0.6451**
Over-sampling	CNN	non-depression	0.5831	0.5565	0.7832	0.6507
		depression		0.6443	0.3862	0.4829
	BiLSTM	non-depression	0.5638	0.6070	0.3581	0.4504
		depression		0.5438	0.7865	**0.6451**
	BERT	non-depression	0.5000	0.0000	0.0000	0.0000
		depression		0.5000	1.0000	0.6667

Bold values represent the greatest levels.

### Result

**Fig. 3:**
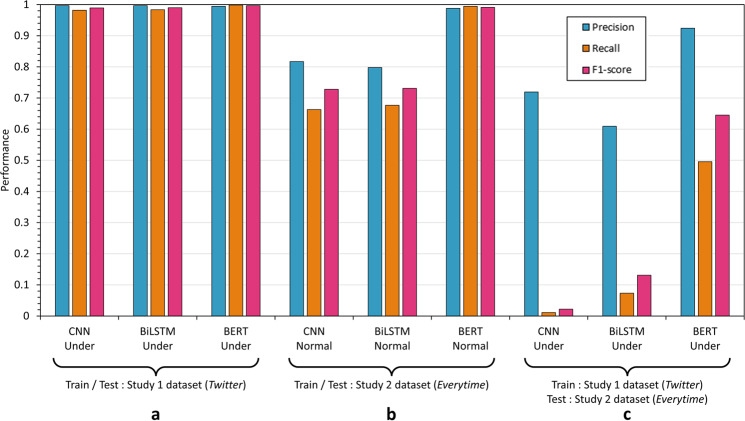
Summary of the results. **a** Korean *Twitter* dataset (Study 1). **b** *Everytime* dataset (Study 2). **c** Trained by Korean *Twitter* dataset and tested by *Everytime* dataset.

To explore the potentiality of our proposed frameworks, we trained and tested our frameworks with *Everytime* dataset. We also compared the results of Study 1 and Study 2. The left and middle of Fig. 3 present the results of Study 1 and 2, respectively. Compared to the results in Study 1, the BERT-based classification shows the great precision 0.9879, recall 0.9945, and F1-score 0.9912, with *Everytime* dataset (Study 2).

Moreover, to address the generalizability of the *Twitter* dataset employed in Study 1 to the depression detection on other platforms, we tested the classifier, trained with the *Twitter* dataset, to the *Everytime* dataset. As presented on the right of Fig. 3, the BERT-based classification model reported the highest F1-score (0.6451) compared to the other baseline models.

Table 7 reports the results of the classification model that was trained with the *Twitter* dataset and tested with the *Everytime* dataset.

## Discussion

Detecting depression in the early stages is an essential issue in effective depression treatment. However, many people are suffering from depression, but are not aware of the symptoms or not receiving proper treatment due to difficulty in accessing treatment services (Hunt and Eisenberg, 2010). Many studies for early detection of depression to combat the problem are being conducted using social media, but most studies are based on English and researches on low-resource languages are insufficient.

The following implications are introduced based on the lessons we learned. First, we have validated the role of social media in the treatment of depression. It is a well-known phenomenon for people to share their emotions and express their mental health symptoms on social media (Kim, 2022). We verified the importance of social media for public mental health by analyzing data collected from *Twitter* and *Everytime* posts to detect depression. Second, for scholars studying mental health, our model can be used as a research method to detect depression. Of course, there have been cases of analyzing mental health symptoms through social media in the past, but in most cases, it usually deals with classification using English text data with existing machine learning or deep learning models such as SVM (Pirina and Çöltekin, 2018), CNN and GRU (Zogan et al., 2021). Therefore, it is significant that by suggesting new depression detection baseline models, we have devised a way to explore public health problems. In addition, our research has the strength of being practical by using multilingual data. We provided unique depression-related datasets consisting of three languages and showed an outstanding baseline for each language. We introduced a series of depression lexicons for three languages. As we verified whether each keyword is related to depression-by-depression experts, our depression lexicons can be applied to other social media such as *Instagram*, to detect depression.

However, several limitations still remain. Because each depression lexicon can have multiple meanings in social media, (i.e., ‘isolation’ can refer to both emotional isolation and physical isolation) lexicon-based labeling is still not absolute. Even considering such an issue, a lexicon-based labeling is an efficient way to analyze millions of text data on social media. Second, as mentioned above, the performance of the classification model between two different domain communities is low. We predict the reason as the lack of cross-domain features (e.g., demographic information) when we tried to detect depression in two communities with one model. Also, we did not evaluate our model with posts from social media users who are diagnosed with depression. Of course, learning only posts from social media users with symptoms of depression could be a stronger model for us. However, since social media can be anonymous and the constructed identity and characteristics in social media can differ from their reality, it is very difficult to collect and model only such datasets in practice.

In future studies, we can extend our research methods based on these findings. We could apply the cross-lingual method with our multiple languages classification models, which integrate each model into one classification model. The cross-lingual classification model can take advantage of high-resource languages (e.g., English). As there are various activities and contents in social media, we plan to develop a user-level multimodal depression detection model using post behavior, image, sentiment, etc rather than simply using text. Additionally, there is a way to expand our findings more broadly. We mainly collected data from anonymous users and students, but in the future, we can measure depression in different and specific age groups. Also, mental health problems are not limited to depression. Thus, we hope that our model can be applied not only to depression but also to other fields of mental health such as anxiety, bipolar, and schizophrenia.

## Supplementary information

Supplemental Material

## Data Availability

The datasets generated during and/or analyzed during the current study are available in the repository, https://github.com/dxlabskku/Mental-Health.

## Footnotes

1 https://www.campuspick.com/.

2 https://everytime.kr/.
