# Supplementary material for: A lexicon-based approach to examine depression detection in social media: the case of Twitter and university community
Source: Humanit Soc Sci Commun. 2022 Sep 21;9(1):325. doi: 10.1057/s41599-022-01313-2 (PMC9491270; doi:10.1057/s41599-022-01313-2)
Supplement: Supplementary file 1 — Supplemental Material [file 41599_2022_1313_MOESM1_ESM.docx]

Supplemental Material associated with:

**A lexicon-based approach to examine depression detection in social media: the case of Twitter and university community**

Appendix 1: Configurations of our models

Table A1. Description of parameter settings used in the experiments

| Sampling | Model | Language | Parameter values |
| --- | --- | --- | --- |
|  |  |  |  |
| Base |  |  |  |
|  | CNN |  |  |
|  |  | Korean | Epochs: 20, Batch size: 64, Learning rate: ${10}^{-3}$, Dropout rate: 0.25 |
|  |  | English | Epochs: 20, Batch size: 1024, Learning rate: ${10}^{-5}$, Dropout rate: 0.25 |
|  |  | Japanese | Epochs: 20, Batch size: 1024, Learning rate: ${10}^{-5}$, Dropout rate: 0.25 |
|  | BiLSTM |  |  |
|  |  | Korean | Epochs: 5, Batch size: 1024, Learning rate: ${10}^{-3}$, Dropout rate: 0.25 |
|  |  | English | Epochs: 5, Batch size: 1024, Learning rate: ${10}^{-3}$, Dropout rate: 0.25 |
|  |  | Japanese | Epochs: 5, Batch size: 1024, Learning rate: ${10}^{-3}$, Dropout rate: 0.25 |
|  | BERT |  |  |
|  |  | Korean | Epochs: 5, Batch size: 8, Learning rate: 2*${10}^{-5}$ |
|  |  | English | Epochs: 5, Batch size: 16, Learning rate: ${10}^{-6}$ |
|  |  | Japanese | Epochs: 5, Batch size: 128, Learning rate: ${10}^{-4}$ |
| Under-sampling |  |  |  |
|  | CNN |  |  |
|  |  | Korean | Epochs: 20, Batch size: 64, Learning rate: ${10}^{-4}$, Dropout rate: 0.25 |
|  |  | English | Epochs: 20, Batch size: 64, Learning rate: ${10}^{-4}$, Dropout rate: 0.25 |
|  |  | Japanese | Epochs: 20, Batch size: 64, Learning rate: ${10}^{-4}$, Dropout rate: 0.25 |
|  | BiLSTM |  |  |
|  |  | Korean | Epochs: 5, Batch size: 64, Learning rate: ${10}^{-3}$, Dropout rate: 0.5 |
|  |  | English | Epochs: 5, Batch size: 64, Learning rate: ${10}^{-3}$, Dropout rate: 0.5 |
|  |  | Japanese | Epochs: 5, Batch size: 64, Learning rate: ${10}^{-3}$, Dropout rate: 0.5 |
|  | BERT |  |  |
|  |  | Korean | Epochs: 5, Batch size: 8, Learning rate: 2*${10}^{-5}$ |
|  |  | English | Epochs: 5, Batch size: 8, Learning rate: ${10}^{-6}$ |
|  |  | Japanese | Epochs: 5, Batch size: 8, Learning rate: ${10}^{-4}$ |
| Over-sampling |  |  |  |
|  | CNN |  |  |
|  |  | Korean | Epochs: 20, Batch size: 64, Learning rate: ${10}^{-4}$, Dropout rate: 0.5 |
|  |  | English | Epochs: 20, Batch size: 1024, Learning rate: ${10}^{-5}$, Dropout rate: 0.25 |
|  |  | Japanese | Epochs: 20, Batch size: 1024, Learning rate: ${10}^{-3}$, Dropout rate: 0.25 |
|  | BiLSTM |  |  |
|  |  | Korean | Epochs: 5, Batch size: 1024, Learning rate: ${10}^{-3}$, Dropout rate: 0.5 |
|  |  | English | Epochs: 5, Batch size: 1024, Learning rate: ${10}^{-3}$, Dropout rate: 0.5 |
|  |  | Japanese | Epochs: 5, Batch size: 1024, Learning rate: ${10}^{-3}$, Dropout rate: 0.5 |
|  | BERT |  |  |
|  |  | Korean | Epochs: 5, Batch size: 8, Learning rate: 2*${10}^{-5}$ |
|  |  | English | Epochs: 5, Batch size: 8, Learning rate: 2*${10}^{-5}$ |
|  |  | Japanese | Epochs: 5, Batch size: 8, Learning rate: 2*${10}^{-5}$ |
